# Effect of Different Yeasts on the Higher Alcohol Content of Mulberry Wine

**DOI:** 10.3390/foods13121788

**Published:** 2024-06-07

**Authors:** Weijia Lian, Jing Lei, Chen Han, Jiuyun Wu, Zhigang Liu, Wei Liu, Ayijiamali Jiapaer, Hanming Su, Yanjun Xu, Ya Chen, Fengjuan Liu

**Affiliations:** 1 Turpan Institute of Agricultural Science, Xinjiang Academy of Agricultural Science, Turpan 838000, China; lwj1634321773@126.com (W.L.); tlfleijing@126.com (J.L.); hxr20170829@126.com (C.H.); kobewjy@163.com (J.W.); lzg780327@126.com (Z.L.); liuwei1030529064@126.com (W.L.); ayjml618@126.com (A.J.); s1723995042@126.com (H.S.); xyj9636@126.com (Y.X.); 2 Institute of Quality Standards & Testing Technology for Agri-Products, Xinjiang Academy of Agricultural Sciences, Urumqi 830091, China

**Keywords:** mulberry wine, yeast, higher alcohol content

## Abstract

Healthy, nutritious, and delicious mulberry wine is loved by everyone, but there is no specific yeast for mulberry wine. To screen for yeasts with low-yield higher alcohols for the fermentation of mulberry wine, we tested five commonly used commercial yeasts available on the market to ferment mulberry wine. All five yeasts were able to meet the requirements in terms of yeast fermentation capacity, speed, and physical and chemical markers of mulberry wine. The national standards were met by the fermentation requirements and the fermented mulberry wine. We identified yeast DV10 as a yeast with low-yield higher alcohols suitable for mulberry wine fermentation. The total higher alcohol content in fermented mulberry wine was 298 mg/L, which was 41.9% lower than that of fermented mulberry wine with yeast EC118. The contents of 17 free amino acids and five sugars in mulberry juice and five yeast-fermented mulberry wines were tested. The results showed that the higher the amino acid and sugar content in yeast-fermented mulberry wine, the higher the content of higher alcohols produced by fermentation. A correlation analysis performed on each higher alcohol produced when yeast DV10 fermented the mulberry wine indicated decreased sugar and related amino acids. The findings demonstrated a substantial negative correlation among the levels of increased alcohol, decreased sugar, and matching amino acid content. Considering the correlation values among increased alcohol, decreased sugar, and related amino acids, the very slight difference suggests that both sugar anabolism and amino acid catabolism pathways have an equivalent impact on the synthesis of higher alcohols during the fermentation of mulberry wine. These results provide a theoretical basis for reducing the content of higher alcohols in mulberry wines, given the history and foundation for producing mulberry wine.

## 1. Introduction

Fructus Mori (*Morus nigra* L.), also known as mulberry, is the mulberry tree fruit cluster in the Moraceae family. It has a sweet and juicy taste and is one of the fruits that people often consume [1], known as a “third-generation fruit” along with seabuckthorn, raspberry, and others. It is listed by the National Health Commission as one of the “homologous medicinal and edible” agricultural products that are both food and medicine [2]. Turpan, Xinjiang, is an important town on the ancient Silk Road, with a long history of planting mulberry trees. Under long-term arid desert climate conditions, Turpan mulberry fruits exhibit various biological characteristics including resistance to drought and cold [3], rich nutrition, disease prevention and treatment, and delayed aging. It is widely used in the food and pharmaceutical industries. Every year, May is the ripening season for Turpan mulberry fruits in Xinjiang. Turpan mulberry fruits are large in size, thick-fleshed, purple-red in color, and rich in sugar; they mature quickly and are difficult to transport [4]. Mulberries can be used to make delicious and nutritious wine. Mulberry wine is rich in nutrients such as anthocyanins, resveratrol, amino acids, trace elements, and antioxidant components [5,6] and has high nutritional and medicinal value [7] and great market potential.

Higher alcohols are a collective term for monohydric alcohols with more than two carbon atoms [8]. They are an important byproduct of the fermentation process of alcoholic beverages and serve as crucial flavor components in alcoholic beverages [9,10]. Because of the influence of raw materials and production processes, higher alcohols are inevitably produced during the mulberry wine brewing process. The proteins in mulberry wine are hydrolyzed into amino acids, which are then processed by decarboxylase and deaminase secreted by yeast or saccharifying bacteria to produce higher alcohols. The higher alcohols produced can also undergo an esterification reaction with organic acids to produce esters, which render the wine a full-bodied and mellow taste and increase the overall coordination of the wine. There are important flavors and aromatic substances in mulberry wine [11]. However, when the content of higher alcohols is too high, the wine has a bitter, strong, astringent, and spicy taste, which not only affects the quality and flavor of the wine but is also harmful to human health [12]. It can cause congestion in the nervous system of drinkers and, because of slow metabolism and an extended retention time in the human body, can cause people to feel headaches and easily become drunk [10,13], which is commonly referred to as the “headache” phenomenon [14]. In severe cases, it can also cause serious physical damage, such as neurotoxicity and hallucinations [15]. Furthermore, its long-term consumption can also lead to chronic poisoning [16,17,18]. Therefore, it is important to regulate the higher alcohol content during mulberry wine fermentation.

Higher alcohols in wine are produced mainly through two pathways as follows: through the degradation and metabolism of amino acids to produce higher alcohols and through sugar metabolism to synthesize higher alcohols [19,20]. The metabolism of yeast is an important factor affecting the production of higher alcohols, and there are significant differences in the types and quantities of metabolites produced by different yeasts [21]. Therefore, there are differences in the types and quantities of higher alcohols in the fermented mulberry wine of different yeast strains. The selection of yeast directly affects the taste and flavor of mulberry wine and is a key factor in determining the quality of mulberry wine. Currently, there are few yeasts available for mulberry wine on the market, and most winemaking enterprises use wine yeast to ferment mulberry juice [22]. Therefore, we selected five types of commercially available wine yeasts to ferment mulberry juice, detected the content of higher alcohols, reduced sugars, and amino acids during yeast fermentation, and analyzed their correlations. We explored the pathways of higher alcohol formation during mulberry juice fermentation and combined the power of yeast fermentation with physicochemical, chemical, and other indicators to identify suitable yeasts for fermenting mulberry wine.

## 2. Materials and Methods

### 2.1. Materials and Instruments

Fresh mulberry was purchased from a fruit store in Gaochang District, Turpan City, in mid-May 2022. Food-grade potassium metabisulfite was purchased from Xinjiang Dingfeng Import and Export Co., Ltd. (Urumqi, China). The yeasts FC9, EC118, KD, DV10, and K1, were obtained from Shanghai Kangxi Food and Beverage Industry Co., Ltd. (Shanghai, China). White Sugar, was obtained from Xinjiang Futang Sugar Industry Co., Ltd. (Altay, China); Pectinase, was obtained from Shanghai Kangxi Food and Beverage Industry Co., Ltd. (Shanghai, China). Various alcohols, including n-butanol, n-propanol, sec-butanol, isobutanol, isoamyl alcohol, n-pentanol, isoamyl alcohol, and β-phenylethanol, were all chromatographically pure, obtained from Shanghai Ampu Experimental Technology Co., Ltd. (Shanghai, China), with isoamyl alcohol, obtained from Tianjin Kemio Chemical Reagent Co., Ltd. (Tianjin, China). Fructose, glucose, sucrose, maltose, and lactose standards were chromatographically pure and obtained from Shanghai Yuanye Biotechnology Co., Ltd. (Shanghai, China). Amino acids such as glycine, L-tyrosine, L-isoleucine, L-phenylalanine, L-leucine, L-alanine, L-valine, L-methionine, L-lysine, L-proline, L-histidine, L-glutamic acid, L-aspartic acid, L-serine, L-threonine, and L-arginine were chromatographically pure and obtained from Shanghai Ampu Experimental Technology Co., Ltd. (Shanghai, China). L-cysteine was also chromatographically pure and obtained from Shanghai Yuanye Biotechnology Co., Ltd. (Shanghai, China).

The instrument used for the analysis included a 7890B gas chromatograph, equipped with an FID detector (hydrogen flame ion detector), Agilent Technology Co., Ltd. (Santa Clara, CA, USA), and an LA8080 amino acid automatic analyzer (Hitachi High Tech Science, Ltd., Tokyo, Japan). An SPX-150B-Z Biochemical Incubator, from Medical Equipment Factory (Shanghai Boxun Industrial Co., Ltd., Shanghai, China), an electronic analytical balance TP-A500 (HZ Electronics Co., Ltd., Meriden, CT, USA), a ZT/H beating machine (Dongba Stainless Steel Food Machinery Parts Distribution Department, Midong District, Urumqi, China), and a portable pH meter PHB-1 (Chengdu Ruixin Instrument Co., Ltd., Chengdu, China) were used for this study.

### 2.2. Experimental Methods

All moldy or rotten fruits, leaves, and small stones were removed from the purchased mulberries (black mulberry fruit, soluble solid content ≥ 13%). Then, a juicer was used to obtain the juice. It was put in a 1000 L fermentation tank along with 0.05 g/kg sulfur dioxide (converted to the amount of added potassium metabisulfite), 0.02 g/kg pectinase, and 0.30 g/kg yeast (yeast FC9, EC118, KD, DV10, and K1) and fermented at 18–20 °C. Three batches of white sugar were introduced in the proper amount during the fermentation process. Halfway through the fermentation period, the second batch was added, and a third of the batch was added at the beginning. Several yeast strains were used to study the fermentation properties of mulberry juice, and pertinent indications were tested. Every strain of yeast was used to ferment three cans of 1000 L mulberry juice.

### 2.3. Fermentation Characteristics of Different Yeasts

From the time of yeast inoculation, the weight of the fermentation container was measured every 24 h. The daily weight change in each mulberry wine sample was recorded until fermentation was complete, and a curve graph was drawn based on the weight loss of CO_2_ during the alcohol fermentation process for each sample until the weight was <2 g. During fermentation, various issues were recorded simultaneously. The start of fermentation time for each treatment and the end time of alcohol fermentation used the total weight loss of CO_2_ as the fermentation power of the yeast, while the weight loss rate of CO_2_ was used as the fermentation rate of the yeast.

### 2.4. Testing Methods

(1) Physical and chemical indicators: The physicochemical indicators of mulberry wine were tested according to GB/T 15038-2006 General Analysis Methods for Wine and Fruit Wine [23]. Various tests were conducted, including the determination of total sugar and reducing sugar through direct titration, total acid using the indicator method, volatile acids through acid–base titration, alcohol content using an alcohol meter, dry extract with a pyknometer, and sulfur dioxide through acid–base titration.

(2) Higher alcohols: Higher alcohols were evaluated according to GB/T 5009.48-2003 Hygienic Standards for Distilled and Compounded Liquors—Analysis Methods 4.2 Methanol and higher alcohol detection [24], using gas chromatography. The chromatographic column was 2 m long with a 4 mm inner diameter (glass or stainless steel) and the following conditions: fixed phase of GDX-102; 60–80 mesh; gasification chamber temperature of 190 °C; detector temperature of 190 °C; column temperature of 170 °C; carrier gas air flow rate (N_2_) of 40 mL/min; hydrogen (H_2_) flow rate of 40 mL/min; air flow rate of 450 mL/min; and injection volume of 0.5 µL.

(3) Amino acids: An appropriate amount of the sample was weighed and mixed well, followed by the addition of 10–15 mL of 6 mol/L hydrochloric acid solution to a hydrolysis tube. The tube was then placed in a refrigerator for 3 to 5 min, with nitrogen protection; the bottle cap was securely tightened, and the tube was placed in a constant temperature incubator set at 110 ± 1 °C for 22 h for hydrolysis. Subsequently, the tube was removed and allowed to cool to room temperature. The hydrolysis tube was opened, and the hydrolysis solution was filtered into a 50 mL volumetric flask. The hydrolysis tube was then rinsed multiple times with a small amount of water, and the washing solution was transferred to the same 50 mL volumetric flask. Finally, the volume was adjusted to a mark with water and stirred thoroughly. Exactly 1.0 mL of filtrate was transferred into a 15 mL test tube. The pressure was reduced to dry at 40 °C, dissolved in 1.0 mL of sodium citrate buffer solution at pH 2.2, shaken and mixed well, passed through a 0.22 µm filter membrane, and then measured using the machine. The LA8080 amino acid analyzer was used for analysis with the following conditions: sulfonic acid cation exchange column measuring 2.6 mm × 150 mm (Guangdong Wengjiang Chemical Reagent Co., Ltd., Shaoguan, China), detection wavelengths set at 570 nm and 440 nm, an injection volume of 500 μL, and a reaction temperature maintained at 135 ± 5 °C.

(4) Sugar: Approximately one gram of the sample was weighed in a 50 mL volumetric flask and dissolved in 20 mL of water. Next, 1 mL of zinc acetate and potassium ferrocyanide solution was slowly added. Additional water was added to the sample, sonicated for 30 min, and then centrifuged. The resulting supernatant was collected using a membrane and subjected to liquid chromatography. An analysis was carried out using liquid chromatography under the following conditions: an amino column with a length of 250 mm, an inner diameter of 4.6 mm, a particle size of 5 μm, a flow rate of 1 mL/min, a column temperature of 40 °C, a running time of 20 min, and an injection volume of 20 μL.

### 2.5. Data Analysis

Each experiment was repeated three times, and the results were expressed as the mean ± standard deviation. The data were analyzed using Excel 2010 software, followed by significant difference analysis, cluster analysis, and correlation analysis using IBM^®^ SPSS^®^ Statistics version 26.0.0 (Pudong, Shanghai, China). The remaining graphs were prepared using OriginPro 2022 Version 225 (Northampton, MA, USA).

## 3. Results

### 3.1. Analysis of the Fermentation Ability of Different Yeasts

**Figure 1:**
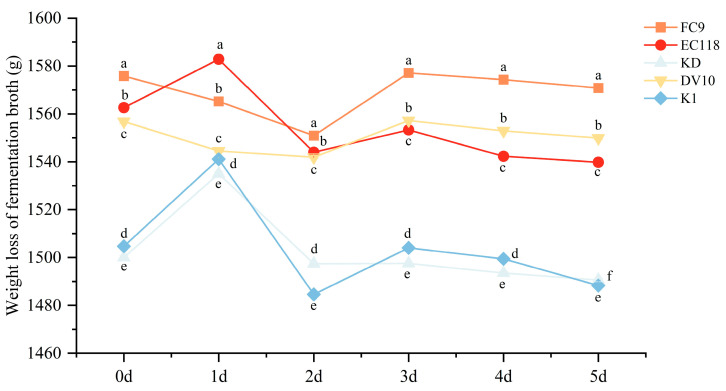
Fermentation power curves for each yeast strain. Note: FC9, EC118, KD, DV10, and K1 represent yeast FC9, yeast EC118, yeast KD, yeast DV10, and yeast K1, respectively. Different lowercase letters within the same column indicate significant differences at *p* < 0.05.

As shown in Figure 1, all five yeast strains began to ferment rapidly after inoculation. At 20 °C, all yeast strains fermented the mulberry juice and reached a peak on the second day. Fermentation was completed on day five. After five days of fermentation, the total weight loss of the mulberry wines fermented with yeasts FC9, EC118, KD, DV10, and K1 was 5 g, 22.8 g, 9.4 g, 6.9 g, and 16.4 g, respectively. By comparing the weight loss of the mulberry wines fermented with five different types of yeast, the starting order of the fermentation strength from strong to weak was as follows: EC1118 > K1 > KD > DV10 > FC9. Based on yeast fermentation experiments, it was concluded that under the conditions of this experiment, yeasts FC9, EC118, KD, DV10, and K1 met the fermentation requirements of mulberry wine in terms of fermentation speed and fermentation ability.

### 3.2. Analysis of the Physical and Chemical Indicators of Mulberry Wine

**Figure 2:**
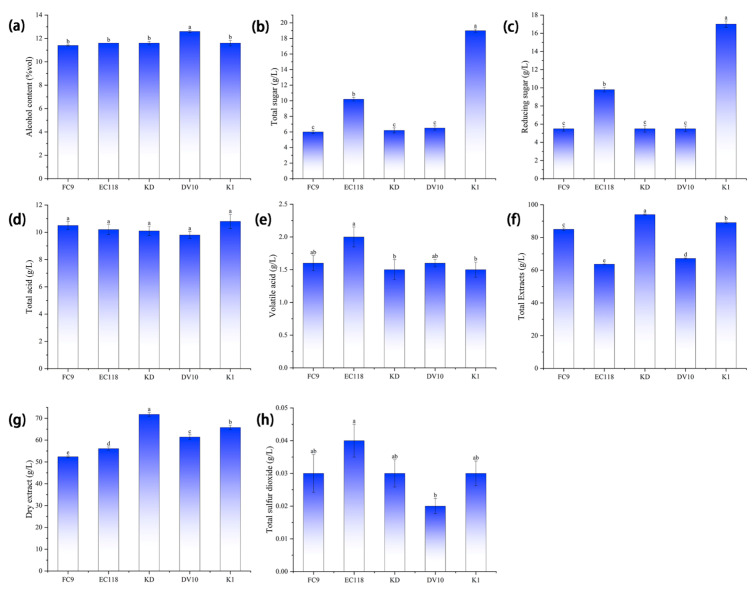
Physical and chemical indicators of mulberry wine fermented with different yeasts. Note: different lowercase letters within the same column indicate significant differences at *p* < 0.05. (**a**) Alcohol content of mulberry wine fermented with five types of yeast; (**b**) total sugar content of mulberry wine fermented with five types of yeast; (**c**) reducing sugar content of mulberry wine fermented with five types of yeast; (**d**) total acid content of mulberry wine fermented with five types of yeast; (**e**) volatile acid content of mulberry wine fermented with five types of yeast; (**f**) total extract content of mulberry wine fermented with five types of yeast; (**g**) content of dry extracts from mulberry wine fermented with five types of yeast; and (**h**) total sulfur dioxide content of mulberry wine fermented with five types of yeast.

As shown in Figure 2, the total sugar and reducing sugar contents of the mulberry wines fermented with the different yeasts were significantly different (*p* < 0.05). The total sugar content of mulberry wine fermented with yeast FC9, EC118, KD, and DV10 met the national standard GB15037-2006 “Wine” [25], which states that the total sugar content of semi-dry wine is >4.0 g/L and <12.0 g/L, indicating a high rate of sugar utilization and thorough fermentation. The total sugar content of the wine fermented by yeast K1 was 19.0 g/L, significantly higher than other treatments (*p* < 0.05), which met the total sugar requirements for semi-sweet wine in GB15037-2006 “Wine” [25]. The sugar utilization rate was not very high, and the fermentation was not sufficiently thorough. The total acid content of the fermented mulberry wine did not differ significantly (*p* > 0.05), and the FC9 and K1 yeasts were 0.6 g/L and 1.0 g/L higher than the average total acid content of the mulberry wine fermented with the other three types of yeast. The appropriate acid content in fruit wine affects the quality, taste, color, and biological stability of the wine and helps inhibit bacteria [26,27]. The volatile acid content of the mulberry wine fermented with different yeasts was the same, with no significant difference (*p* > 0.05), while the volatile acid content of the mulberry wine fermented with yeast EC118 was 0.4 g/L or 0.5 g/L higher than that of the other yeasts. The total and dry extracts of the mulberry wine fermented with the different yeast strains showed significant differences (*p* < 0.05), whereas the sulfur dioxide content of the mulberry wine fermented with the five yeast strains was not significantly different (*p* > 0.05), all of which met the requirements. The difference in the alcohol content of the fermented mulberry wine was not significant (*p* > 0.05). The alcohol content of the mulberry wine fermented with yeast DV10 was 1.2% higher than that fermented with yeast FC9 and 1.0% higher than those fermented with the other three types of yeast. The alcohol-producing ability of DV10 was strong.

### 3.3. Analysis of the Higher Alcohol Content in Mulberry Wine

**Figure 3:**
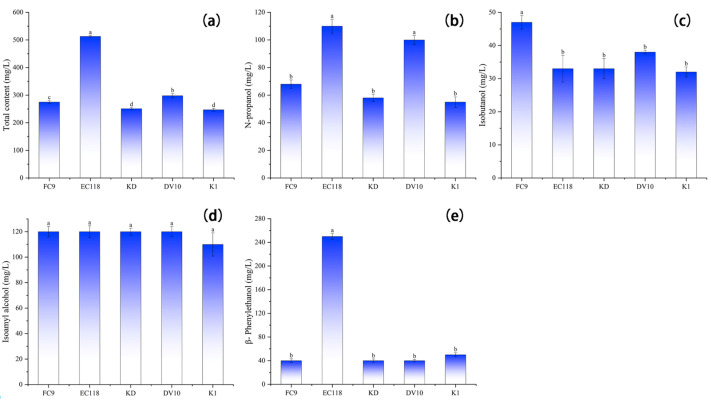
Higher alcohol content of mulberry wine fermented by different yeasts. Note: different lowercase letters within the same column indicate significant differences at *p* < 0.05. (**a**) Total higher alcohol content of mulberry wine fermented with five types of yeast; (**b**) n-propanol content of mulberry wine fermented with five types of yeast; (**c**) isobutanol in mulberry wine fermented with five types of yeast; (**d**) isoamyl alcohol content in mulberry wine fermented with five types of yeast; and (**e**) β-phenylethanol content in mulberry wine fermented with five types yeast. FC9, EC118, KD, DV10, K1 and SS represent yeast FC9, yeast EC118, yeast KD, yeast DV10, yeast K1, and mulberry juice, respectively.

As shown in Figure 3, there was a significant difference in the total content of higher alcohols in the mulberry wine fermented with the different yeasts (*p* < 0.05). The lowest total content of higher alcohols in yeast K1 fermentation was 247 mg/L, which was 10.18%, 51.85%, 1.59%, and 17.11% lower than in yeast FC9, EC118, KD, and DV10 fermentations, respectively. The highest total content of higher alcohols in yeast EC118 was 513 mg/L. The n-butanol, sec-butanol, and n-pentanol contents in the higher alcohols of the mulberry wine fermented with the five types of yeast were below the detection limit (<0.001 mg/L) and were not detected. During the fermentation of mulberry wine with the different yeasts, n-propanol, isobutanol, and β-phenylethanol showed significant differences (*p* < 0.05). The highest β-phenylethanol content in the mulberry wine fermented with yeast EC118 was 250 mg/L, which was 6.25 times higher than yeast FC9, KD, and DV10 and five times higher than yeast K1. There was no significant difference in the isoamyl alcohol content of the mulberry wine fermented with the different yeast strains (*p* > 0.05). The isoamyl alcohol content in the mulberry wine fermented with yeast K1 was 10 mg/L lower than that of the other four yeast strains. Isoamyl alcohol was the highest among the higher alcohol content in mulberry wine.

### 3.4. Analysis of the Free Amino Acid Content in Mulberry Wine

**Figure 4:**
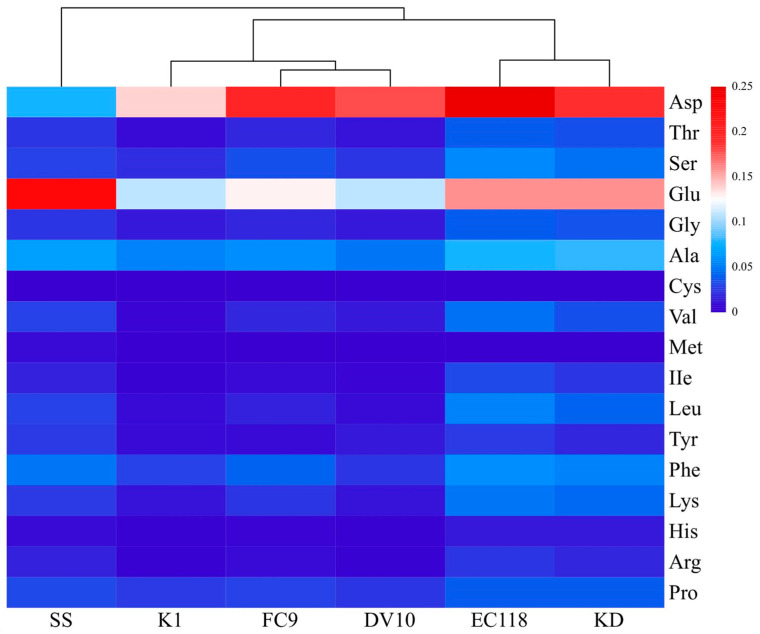
Heat map analysis of amino acid content in mulberry juice and wine. Note: Asp = aspartic acid; Thr = threonine; Ser = serine; Glu = glutamic acid; Gly = glycine; Ala = alanine; Cys = cysteine; Val = valine; Met = methionine; IIe = isoleucine; Leu = leucine; Tyr = tyrosine; Phe = phenylalanine; Lys = lysine; His = histidine; Arg = argnine; Pro = proline. FC9, EC118, KD, DV10, K1, and SS represent yeast FC9, yeast EC118, yeast KD, yeast DV10, yeast K1 and mulberry juice, respectively.

The main hydrolyzed amino acids in the mulberry wine fermented with the five types of yeast were aspartic acid, glutamic acid, and alanine. These three main amino acids accounted for 62.03%, 50.51%, 51.50%, 67.65%, and 67.33% of the total amino acid content, respectively. According to the analysis of the content of each amino acid in the clustering heatmap in Figure 4, the amino acids in the mulberry juice and wine fermented with the five types of yeast exhibited certain clustering characteristics and were clustered into three categories as follows: the first category included EC118 and KD, with the closest and highest amino acid content, respectively. The contents of threonine, glycine, alanine, valine, isoleucine, leucine, phenylalanine, lysine, histidine, arginine, and proline were higher than the corresponding amino acid content in the mulberry juice. The second category included K1, FC9, and DV10, which had relatively low levels of amino acids. Total amino acid content was ranked FC9 > mulberry juice > DV10 > K1. The third type was mulberry juice, with most of its 17 amino acids falling between those of the first and second types. The glutamic acid, aspartic acid, and alanine contents in the mulberry juice were 0.23, 0.080, and 0.066 g/100 g, respectively, representing 53.02% of the total hydrolyzed amino acid content. The order of essential and non-essential amino acids was EC118 > KD > mulberry juice > FC9 > DV10 > K1. Yeasts with a higher amino acid content after fermentation produced higher alcohols.

### 3.5. Analysis of the Sugar Content in Mulberry Wine

**Figure 5:**
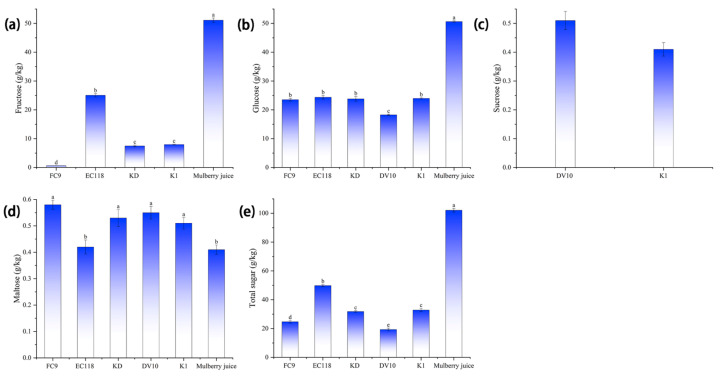
Sugar content in the mulberry wine fermented with different yeasts. Note: different lowercase letters within the same column indicate significant differences at *p* < 0.05. (**a**) Fructose content of mulberry wine and mulberry juice fermented by four types of yeast; (**b**) glucose content of mulberry wine and mulberry juice fermented by five types of yeast; (**c**) sucrose content of mulberry wine fermented by two types of yeast; (**d**) maltose content of mulberry wine and mulberry juice fermented by five types of yeast; and (**e**) total sugar content of mulberry wine and mulberry juice fermented by five types of yeast.

Figure 5 shows that there were significant differences (*p* < 0.05) in the fructose, glucose, sucrose, and maltose content in the five yeast-fermented mulberry wines. The total sugar content was in the following order: mulberry juice > EC118 > K1 > KD > FC9 > DV10, and the fructose and glucose contents in mulberry wine were lower than those of mulberry juice. The sucrose content in mulberry wine fermented with yeasts DV10 and K1 was 0.51 and 0.41 g/L, respectively. The sucrose content in the mulberry wines fermented with the other three types of yeast was not detected (<0.20 g/L). The maltose content in the mulberry wine fermented with the five types of yeast was higher than that of the mulberry juice. Yeasts with a high sugar content produced more higher alcohols after fermentation. The yeasts showed different utilization rates of sugar, resulting in the different types and contents of sugars in the wine after fermentation.

### 3.6. Analysis of Changes in Reducing Sugar and Higher Alcohol Content during Mulberry Wine Fermentation

Most people agree that wine can have a pleasant flavor without having any negative effects when its total alcohol content exceeds 300 mg/L. The most appropriate higher alcohol percentage for the mulberry wine fermented by yeast DV10 is 298 mg/L, as indicated by the findings of 3.3. Consequently, yeast DV10 was selected to ferment mulberry wine, and during the fermentation process, changes in higher alcohols and components that influence the higher alcohol content were observed and analyzed.

**Figure 6:**
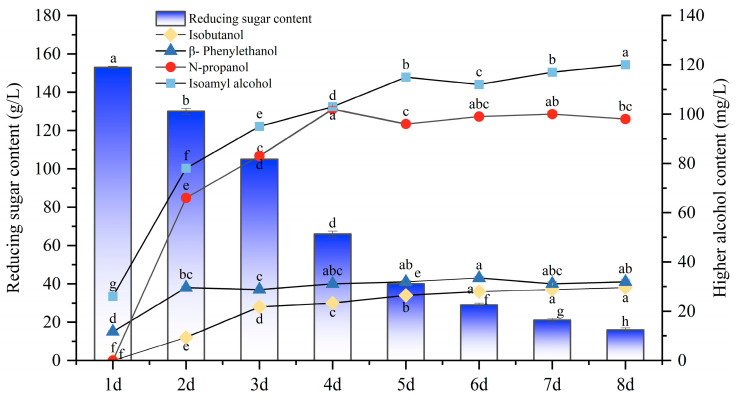
Changes in reducing sugar content and higher alcohol content during the fermentation process of mulberry wine. Note: different lowercase letters within the same column indicate significant differences at *p* < 0.05.

Figure 6 shows that during the fermentation of mulberry wine with yeast DV10, the reduced sugar content continuously decreased from 153 to 16 g/L, which is consistent with the law of yeast fermentation using reducing sugars to produce alcohol. Isopentanol had the highest content among the four highest alcohols during fermentation, showing an overall increasing trend. On the second day of fermentation, the isopentanol content was twice that on the first day. On the eighth day of fermentation, the highest isopentanol content was 120 mg/L. N-propanol was the second highest of the four higher alcohols in the fermentation process, which generally increased first and then decreased, with the highest content reaching 102 mg/L on the fourth day of fermentation. β-phenylethanol was the third highest among the four higher alcohols in the fermentation process, showing an overall trend of increasing first and then remaining stable. The content increased the most on the second day, with an increase of 23 mg/L, reaching its highest value of 43 mg/L on the sixth day of fermentation. Isobutanol was the lowest of the four higher alcohols in the fermentation process, showing an overall upward trend, with the highest content reaching 38 mg/L on the eighth day of fermentation. The amount of higher alcohols synthesized as a consequence of yeast metabolism is closely correlated with the rate at which yeast grows. Following the addition of yeast to mulberry juice, the yeast grows more quickly and has a rapid metabolism. Yeasts can extract carbon fragments from the metabolism of sugar, primarily α-ketonic acid, which creates higher alcohols produced by various strains of yeast. Variations in their physical characteristics result in variations in the kinds and amounts of higher alcohols that their metabolism produces.

### 3.7. Analysis of Changes in Amino Acid Content and Higher Alcohol Content during Mulberry Wine Fermentation

**Figure 7:**
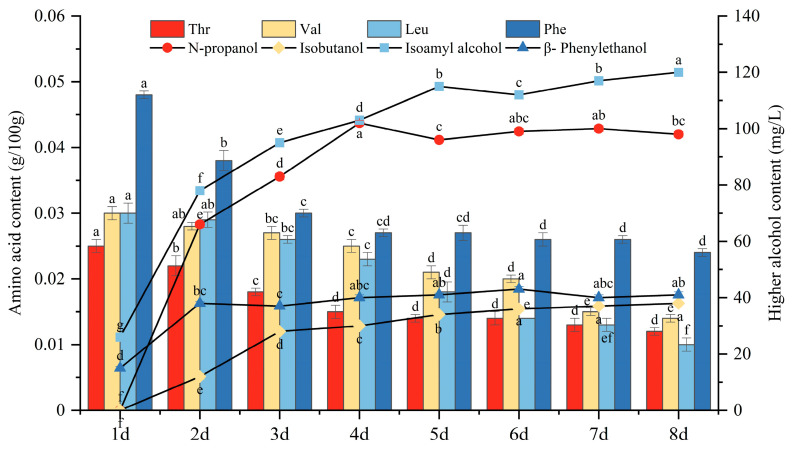
Changes in amino acid and higher alcohol content during mulberry wine fermentation. Note: different lowercase letters within the same column indicate a significant difference at *p* < 0.05.

Figure 7 shows that during the fermentation of mulberry wine with yeast DV10, the leucine content continued to decrease, and the corresponding isoamyl alcohol content was the highest among the four higher alcohols in the mulberry wine fermentation process. The valine content was higher than that of leucine and continued to decrease during fermentation, resulting in the lowest isobutanol content among the four highest alcohols. During fermentation, threonine also showed an overall decreasing trend, with amino acids having the lowest content but the smallest decrease among the four amino acids. The corresponding content of n-propanol produced was the highest among the four higher alcohols, second only to that of isoamyl alcohol. Phenylalanine was the amino acid with the highest content and the highest decrease among the four amino acids. Overall, it also showed a decreasing trend, with a maximum decrease of 0.01 g/100 g on the second day. The corresponding phenylethanol content initially increased and then remained constant.

### 3.8. Correlation Analysis of Higher Alcohols, Amino Acids, and Reducing Sugars during the Fermentation of Mulberry Wine

**Figure 8:**
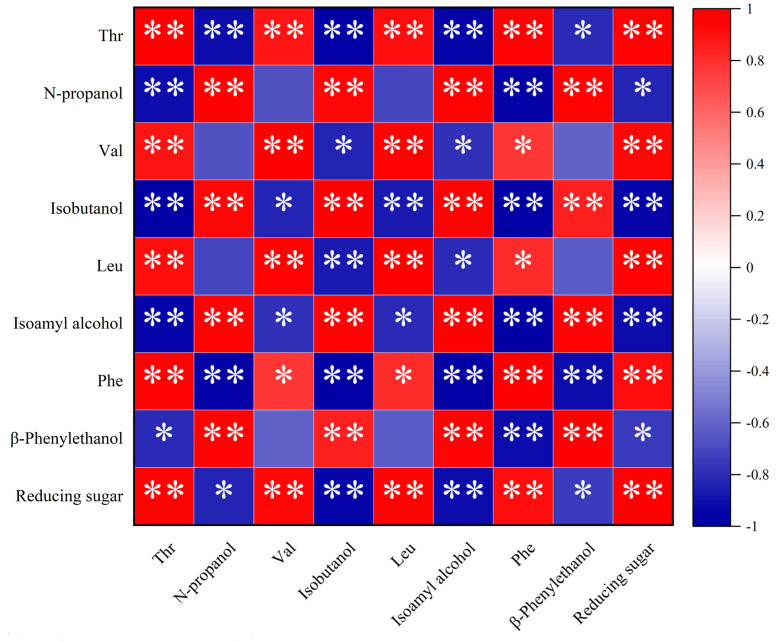
Correlation analysis of higher alcohols, amino acids, and reducing sugars during the fermentation of mulberry wine. Note: * *p* ≤ 0.05, ** *p* ≤ 0.01.

A correlation analysis was conducted for various higher alcohols, reducing sugars, and amino acids during the fermentation of mulberry wine with yeast DV10, as shown in Figure 8. The corresponding amino acids were threonine, corresponding to n-propanol; valine, corresponding to isobutanol; leucine, corresponding to isopentanol; and phenylalanine, corresponding to β-phenylethanol. The results in Figure 4 show that the content of higher alcohols was significantly negatively correlated with the content of reducing sugars and the corresponding amino acid content. Furthermore, the correlation coefficients among higher alcohols, reducing sugars, and corresponding amino acids were very small, indicating that higher alcohol synthesis in mulberry wine fermentation was influenced by both the amino acid decomposition and sugar synthesis metabolism pathways, and both pathways contribute equally.

## 4. Discussion

The contributions of amino acid catabolism and sugar synthesis metabolism to the formation of higher alcohols are different and influenced by the composition and content of the nitrogen sources available in the culture medium. The final production of higher alcohols is the result of a gradual balance between the two metabolic pathways, with an increase in available nitrogen sources in the culture medium [28]. Giudici P [29] proposed and demonstrated that both pathways are equally important for the generation of higher alcohols. Guymon J F [30] proposed that 75% of the total amount of isobutanol, isoamyl alcohol, and 2-methyl-1-butanol in wine comes from the amino acid catabolism pathway and 25% comes from the sugar synthesis metabolism pathway. Yunping Wei [31] proposed that the formation of higher alcohols during the fermentation process of kiwifruit wine is closely related to sugar metabolism, and its production may be greatly influenced by the synthetic metabolic pathway. This study analyzed the correlation among higher alcohols, amino acids, and reducing sugars during mulberry wine fermentation. The two pathways contributed equally to the production of higher alcohols in mulberry wine. Therefore, two approaches should be considered to reduce the higher alcohol content in mulberry wine. The first is the breeding of excellent yeast strains for the brewing of higher alcohol with low yield, which can fundamentally reduce the yield of higher alcohols, mainly through mutagenic breeding and genetic engineering breeding [32]. In this experiment, no yeast strains were selected, but two yeast strains with a lower alcohol content suitable for fermenting mulberry wine were selected from five commercial yeast strains. The second is to start from the fermentation process conditions, control the fermentation temperature, pH, amount of inoculation, and other factors, and reduce the content of higher alcohols in the wine [32]. The next step will be to carry out this work.

## 5. Conclusions

To screen for suitable yeast strains for mulberry wine fermentation, we selected five commonly used commercial yeast strains available on the market to ferment mulberry wine. The content of higher alcohols in the fermented mulberry wine was measured, along with the levels of n-propanol, isobutanol, and β-phenylethanol. There was a significant difference in the content of phenylethanol (*p* < 0.05), and isoamyl alcohol was the predominant higher alcohol in the mulberry wine. The higher alcohol content in the mulberry wine fermented with different yeasts was in the following order: EC118 > DV10 > FC9 > KD > K1. The total higher alcohol content in the wines fermented by yeasts DV10 and FC9 was close to 300 mg/L, which is an appropriate higher alcohol content.

The contents of 17 free amino acids and five sugars in the mulberry juice and five yeast-fermented mulberry wines were detected separately. The results showed that there were significant differences in the total amino acid, essential amino acid, and non-essential amino acid content of the mulberry wine fermented with the different yeasts (*p* < 0.05). The total amino acid content of the fermented mulberry wine was EC118 > FC9 > KD > mulberry juice > DV10 > K1, and the essential amino acid and non-essential amino acid contents were EC118 > KD > mulberry juice > FC9 > DV10 > K1. The cluster analysis divided the types and contents of amino acids in mulberry juice and the five types of yeast-fermented mulberry wine into four categories. Mulberry juice belonged to one category; yeasts KD and EC118 belonged to one category; yeast K1 belonged to one category; and yeasts DV10 and FC9 belonged to one category. The contents of fructose, glucose, sucrose, and maltose in the mulberry wine fermented with the different yeasts were significantly different (*p* < 0.05). The total sugar content decreased in the following order: mulberry juice > EC118 > K1 > KD > FC9 > DV10. The higher the amino acid and sugar content of the mulberry wine fermented with the five types of yeast, the higher the content of higher alcohols produced by fermentation. A correlation analysis was performed for various higher alcohols, reducing sugars, and corresponding amino acids during the fermentation of mulberry wine with yeast DV10. The results showed that the content of higher alcohols was significantly negatively correlated with the content of reducing sugars and corresponding amino acids. The correlation coefficients among higher alcohols, reducing sugars, and corresponding amino acids were very small, indicating that the synthesis of higher alcohols in mulberry wine fermentation is influenced by both amino acid decomposition and sugar synthesis metabolism, and the contributions of the two pathways are the same. These results provide a theoretical basis and data support for reducing the content of higher alcohols in mulberry wine and optimizing the brewing process.

By comparing and analyzing the fermentation performance of physicochemical and chemical indicators, five types of yeast (FC9, EC118, KD, DV10, and K1) met the fermentation requirements of mulberry wine in terms of fermentation speed and fermentation ability. DV10 yeast had a total content of higher alcohols (298 mg/L) in fermented mulberry wine, which was 41.9% lower than in the mulberry wine fermented with yeast EC118. After drinking mulberry wine, it is rare to have headaches and dizziness. It is a suitable commercial yeast for brewing mulberry wine and provides a reference for mulberry wine production.

## Data Availability

The original contributions presented in this study are included in this article. Further inquiries can be directed to the corresponding author.
